# Differential Predictive Effect of Self-Regulation Behavior and the Combination of Self- vs. External Regulation Behavior on Executive Dysfunctions and Emotion Regulation Difficulties, in University Students

**DOI:** 10.3389/fpsyg.2022.876292

**Published:** 2022-06-22

**Authors:** Jesús de la Fuente, José Manuel Martínez-Vicente, Mónica Pachón-Basallo, Francisco Javier Peralta-Sánchez, Manuel Mariano Vera-Martínez, Magdalena P. Andrés-Romero

**Affiliations:** ^1^Department of Theory and Methods of Educational and Psychological Research, School of Education and Psychology, University of Navarra, Pamplona, Spain; ^2^Department of Psychology, School of Psychology, University of Almería, Almería, Spain; ^3^Department of Psychology, School of Psychology, University of Granada, Granada, Spain

**Keywords:** executive functions, self-regulation, self- vs. external regulation, emotion regulation difficulties, university students

## Abstract

The aim of this research was to establish linear relations (association and prediction) and inferential relations between three constructs at different levels of psychological research – *executive dysfunction* (microanalysis), *self-regulation* (molecular level), and *self-* vs. *external regulation* (molar level), in the prediction of emotion regulation difficulties. We hypothesized that personal and contextual regulatory factors would be negatively related to levels of executive dysfunction and emotion regulation difficulties; by way of complement, non-regulatory and dysregulatory personal, and contextual factors would be positively related to these same difficulties. To establish relationships, we used a retrospective, *ex post* facto design, where 298 university students voluntarily participated by completing standardized self-reports. Linear and structural correlational, predictive analyses were performed, as well as inferential analyses. Results were consistent and validated the proposed hypotheses, for both association and prediction. The most important result refers to the discriminant value of the five-level combination heuristic for predicting Executive Function and External (contextual) Dys-Regulation. In conclusion: (1) both personal and contextual regulation factors must be analyzed in order to better understand the variation in executive functions and emotion regulation difficulties; (2) it is important to continue connecting the different levels of the constructs referring to self-regulation, given their complementary role in the behavioral analysis of regulation difficulties.

## Introduction

Self-regulatory behavior as a behavioral meta-skill is exclusive and inherent to human beings. Given its importance, it has been a classic study variable in the realm of psychology (Baumeister and Heatherton, 1996). From the perspective of the evolution of our species, this behavior has come about thanks to the development and plasticity of the prefrontal area of the brain, as has been amply demonstrated in phylogenetic, ontogenetic, neurological, and psychological research (Bull and Espy, 2006; Zelazo and Carlson, 2012; Friedman and Robbins, 2022). In fact, exploration of missing or delayed prefrontal development, characteristic of executive dysfunctions, has helped us understand the biological basis for the self-regulation process (Gioia et al., 2000a). This superior neurological foundation, however, with its associated cognitive and emotional processes, does not *per se* guarantee adaptability and success in the behavioral execution of different human tasks. Numerous contextualized learning and relearning experiences are required throughout life, in different adaptive situations, for this meta-skill to reach an optimal level of development (Duckworth and Carlson, 2013). Such training results from exposure to appropriate experiences, models and contingencies that promote a complete, rich behavioral repertoire.

From all the foregoing, we infer the need: (1) to investigate the relationship between different constructs used in reference to self-regulation – constructs which come about at different levels of psychological analysis; (2) to precisely define such relationships, as well as the summative or interactive effects of the different variables involved, in order to more accurately identify the contextual factors of executive function (EF).

### Different Levels of Psychological Analysis of Regulatory Constructs

The evolution of research on behavioral self-regulation has led to different types and subtypes of psychological constructs that seek to explain this type of behavior in a given research domain (de la Fuente et al., 2019a,b).

#### Level of Microanalysis: Executive Functions as a Neurological Construct (Neuropsychological Level)

The most basic level of behavioral analysis, called microanalysis, or the neuropsychological level, has suggested the concept of EFs to refer to the neurological foundation of self-regulation (de la Fuente et al., 2019a). The term “executive functions” is a many-faceted concept, often considered an “umbrella term” that encompasses a group of processes, interrelated among themselves, and responsible for “guiding, directing, and controlling cognitive, emotional, and behavioral functions, especially during the active solution of novel problems” (Gioia et al., 2000b). It is a broad construct that encompasses both goal-directed processes (Knight and Stuss, 2002) and the emotion and behavior regulation necessary to adapt to the environment where the goals are to be achieved (Bechara et al., 2000; Stuss and Alexander, 2000). The study of EFs presents significant difficulties (Levy and Anderson, 2008; Anderson and Reidy, 2012), such as lack of consensus on its definition and the emergence of multiple models. There is no single definition for EFs; on the contrary, nearly every model proposed involves a different conceptualization.

Executive function is commonly conceptualized in terms of cognitive processes that enable future goal-directed behaviors, and involves the processes of planning, organization, inhibitory control, cognitive flexibility, and problem solving (Carlson et al., 2013; Diamond, 2013). Zelazo and Carlson (2012) consider EFs to be comparable to cognitive control; they refer to “top-down neurocognitive processes involved in the conscious, goal-directed control of thought, action, and emotions.” The main components are cognitive flexibility, inhibitory control, and working memory (Miller and Cohen, 2001; Miller and Wallis, 2009). This construction has been used to evaluate the learner’s discrete cognitive behaviors, such as executive memory, attention, behavior inhibition, and processing speed. This construct is currently producing a large quantity of evidence, making it possible to explain learning problems associated with neurological deficits (Friedman and Robbins, 2022). In recent years, aspects such as theory of mind, emotion regulation, empathy, and the affective aspects of decision making have been included as the so-called “warm” or “hot” EFs (Gioia et al., 2000a; Mesulam, 2002, Tranel, 2002; Eslinger et al., 2004; Happaney et al., 2004; Kerr and Zelazo, 2004; Stuss and Anderson, 2004). The term “cool” EFs is thus reserved for the more cognitive aspects that have been studied traditionally (Zelazo et al., 2005).

Despite its relevant contributions, this construct is specific to the clinical or neurological field, and places the contextual variables of human learning at a distal level of analysis. It is reasonable to assume that this construct, as a neuropsychological substratum of self-regulation, is related to and associated with other personal characteristics at the molecular level, worth being considered as such. In this regard, relations between EF and personality have been found. Greater difficulty in subjective EF was registered by older adults with greater negative affect, and by older adults higher in neuroticism and lower in conscientiousness (Bell et al., 2020). As a complement, there is growing evidence that addresses cognitive deficits in EF. A relationship between EF and Borderline Personality Disorder (BPD) has long been suggested by evidence of high comorbidity between BPD and disorders characterized by poor EF, for example, attention deficit/hyperactivity disorder, ADHD (MccLure et al., 2015; Bell et al., 2020). However, recent studies have also documented prevalence in association with socioeconomic and cultural level (Fayyad et al., 2017), contextual aspects that are yet to be accurately defined. This study reported that the current prevalence of ADHD in DSM-IV/CIDI adults averaged 2.8% across all surveys and was higher in countries with high income (3.6%) or upper middle income (3.0%), than in low-income/lower-middle-income (1.4%) countries. ADHD in adults is significantly related to being male, having a previous marriage, and low education. Adult ADHD had high comorbidity with DSM-IV/CIDI disorders of anxiety, mood, behavior, and substance use; and was significantly associated with role impairments (days out of role, cognitive impairment, and social interactions), when controlling for comorbidities.

#### Molecular Level of Analysis: Self-Regulation Behavior (Personal and Clinical Level)

In a complementary approach, research has established self-regulation as an essential meta-behavioral skill, which guides the learning process (de la Fuente, 2017). Self-regulation is thus trainable, and is exclusive to human beings. The bidirectional relationship between EFs and self-regulation (SR) has been established in different contexts, such as in the level of childhood development (Finders et al., 2021), eating behavior (Hawkins et al., 2021), and persistent effort (Barkley, 2021). Some models of EFs have even centered on self-regulation (Granziera et al., 2021).

Also, from an eminently psychoeducational approach, the abundant prior research based on the model Zimmerman and Schunk (2001) has operationally specified the behaviors typical at each sequential phase of human learning (before, during, and after). Despite its goodness, however, this model belongs to a molecular level of analysis, and so does not rule out possibilities for investigation at other levels (de la Fuente et al., 2019a).

#### Analysis of Self-Regulation at the Molar Level: Self- vs. External Regulation Behavior (Personal and Contextual Level)

At the level of molar analysis, which is more interactive and context-oriented, a comprehensive model has been proposed that allows us to understand personal regulatory factors in interaction with the context. Only in this way, it is assumed, can teaching-learning processes be evaluated in real contexts and not only in the laboratory. In this line, evolving research has led to the proposal of two behavioral constructs, represented in the theory of self-regulated vs. externally regulated learning, or SRL vs. ERL Theory (de la Fuente, 2017; de la Fuente et al., 2019b,2020a,b,2021a).

First, a possible gradation of regulation levels has been established, for both the individual and their context. For the individual, a progressive range of regulatory behavior has been defined: Self-Regulation (SR) vs. Non-Regulation (NR) vs. Dys-Regulation (DR). Self-regulation (SR) would be characterized by an adequate level of skill and high execution; Non-Regulation (NR) would be characterized by a medium level of the former, or by behavior bereft of regulatory effort; finally, Dys-Regulation (DR), would be characterized by a low level of regulation, along with execution of maladaptive regulatory behaviors, such as behavioral excesses or deficits. Evidence has shown that self-regulation correlates negatively with non-regulation, while non-regulation correlates positively with dysregulation. This is to say, when a person stops making regulatory effort, they are more likely to ultimately develop dysregulatory behavior. This schema is applicable to behaviors in education and health.

Secondly, regulatory levels pertaining to the context have been defined. As in the former case, a progressive range of regulation contexts has been identified: Externally Regulatory (ER) vs. Externally Non-Regulatory (ENR) vs. Externally Dys-Regulatory (EDR). A regulatory context (ER) would be characterized by adequately promoting the individual’s self-regulation, by means of helps, indications, or external contingencies to induce high execution of SR behavior. An externally non-regulatory (ENR) context would be characterized by a medium presence of external regulation, in other words, inconsistent promotion of self-regulation, leaving regulatory effort up to the individual. Finally, an externally dysregulatory context (EDR) would be characterized by actively promoting dysregulation in the individual, by means of negative modeling, inappropriate indications, and/or erroneous contingencies, that actively encourage behavioral excesses or deficits. The evidence in this aspect has shown that externally regulatory contexts encourage self-regulation, while non-regulatory contexts promote non-regulation and dysregulatory contexts promote dysregulation. Moreover – and most importantly – a regulatory context decreases the likelihood of a non-regulatory context, but a non-regulatory context increases the probability of a dysregulatory context. Preliminary research in the areas of education and health has shown that a dysregulatory context promotes dysregulatory behavior. Thus, when a teaching process is dysregulatory, students learn more poorly, and use poorer self-regulation strategies (de la Fuente et al., 2019b,2020a,2021b). A dysregulatory health context, in similar fashion, positively predicts more reactance behaviors and the practice of poorer health behaviors (Pachón-Basallo et al., 2021).

Third, we have considered the regulation factors from a joint or combined analysis: (1) the level of internal regulation: personal self-regulation; (2) the level of external regulation: regulation promoted by the context; (3) the possible interactions between the two. These types of interaction have been identified in a five-level heuristic. Different teaching-learning processes have been intensively analyzed and the goodness of the proposal has been empirically verified. Academic achievement, learning approaches, procrastination, student engagement, and motivational-affective variables have been shown to be dependent on this interactive combination of Personal factors × Contextual factors. The focus is not exclusively on students’ individual variables, as in previous (mainly molecular-level) research, but also on the Learning × Teaching interaction, having a more molar nature. This information is very important in helping to conceptualize learning behavior from a broader view, not only from discrete cognitive processes, such as the regulatory behaviors of students. Prior evidence has shown that all combinations of the cognitive and emotional variables are observed (de la Fuente et al., 2019b,2020b,2021b).

### Emotion Regulation Difficulties

In order to accomplish one’s goals, emotions must be regulated through the use of intrinsic and extrinsic processes that monitor, assess, and adapt one’s emotional reactions as needed (Thompson, 1994). This idea of emotion regulation assumes that emotions are functional, giving us information about our context and prompting behaviors that can help us adapt to situational demands (Izard and Ackerman, 2000). By contrast, if there is a deficit in awareness, understanding, or modulation of one’s emotions, adaptation becomes more difficult and this may lead to negative outcomes in many different ways. More and more research is showing the role of emotion regulation difficulties in many types of psychopathology and maladaptive behaviors (Gross and Jazaieri, 2014; Sheppes et al., 2015). Self-report measures that assess emotion regulation have thus become a priority in the clinical approach to emotion regulation, and many new instruments have been developed and validated. Dimensions of emotion regulation difficulties (ERD) (e.g., emotional non-acceptance, lack of emotional awareness, and clarity) and maladaptive strategies for regulating emotions (e.g., avoidance and suppression) are addressed in a number of empirically supported measures. One prominent scale in the scientific literature is the *Difficulties in Emotion Regulation* Scale (DERS; Gratz and Roemer, 2004, 2008; Gratz and Tull, 2010), which measures a broad range of emotion regulation difficulties. ERD is considered a multidimensional construct, consisting of a set of behaviors that range from lack of self-knowledge and awareness of one’s emotions, to difficulty in managing them. Emotion regulation difficulties have been related to different adaptive issues, such as the use of technology devices (Horwood and Anglim, 2021), food and substance abuse (Barnhart et al., 2021), health-related behavior (Lewczuk et al., 2021), and psychopathological symptoms of depression (Melero et al., 2021).

### Objectives and Hypotheses

Based on prior evidence, this study seeks to confirm the associations, predictions, and interdependence relations between the three levels of the constructs cited above, in order to establish their relationship to emotion regulation difficulties. Different types of hypotheses were posed:

*Association hypothesis.* (1) A significant negative association is expected between the molecular construct SR and the microanalysis construct of executive dysfunction. However, in the case of the molar construct Self-Regulation (SR)/External Regulation (ER), while the expected relationship is positive and significant for SR and ER, it is negative for Non-Regulation (NR), Dys-Regulation (DR), External Non-Regulation (ENR), and External Dys-Regulation (EDR). We also expect a positive association relationship between difficulties in EFs and emotion regulation difficulties (ERD).

*Prediction hypothesis.* (2) The components of SR will prove to be negative predictors of the EF score. SR-ER factors should prove to be differential predictors of EF: while internal and external regulatory factors (SR-ER) should be positive predictors, internal and external non-regulatory or dysregulatory factors (NR, DR, ENR, and EDR) should be negative. Finally, EF difficulties will be positive predictors of emotion regulation difficulties.

*Structural prediction hypothesis.* (3) The combined level of internal and external regulation (SR-ER) will be a strong predictor of EF, differentially and significantly, as will SR alone, to a lesser degree. SR-ER will be a positive predictor; NR-ENR and DR-EDR, negative predictors. EF difficulties positively predict emotion regulation difficulties.

*Inferential hypothesis.* (4) EF levels (low–medium–high) will positively determine levels of SR and ER, and differentially determine levels of SR-ER (positively) and NR-ENR, DR-EDR (negatively). In complementary fashion, the five combination levels of internal and external regulation (SR-ER) will be significant, negative determinants of EF and the degree of emotion regulation difficulties, though differentially. Combined low levels of SR-ER will determine higher levels of EF difficulties and EDR, and vice versa, in gradient manner.

## Materials and Methods

### Participants

The study sample contained a total of 298 undergraduate students from 15 different degree programs enrolled in Spanish or Latin American universities. The students were pursuing degrees in Psychology, Primary Education, or Educational Psychology; 63.5% were female and 36.5% were male. Students’ age fell between 19 and 25, with a mean age of 23.12 years (SD = 2.679). The study design was incidental and non-randomized. As an inclusion criteria, university degree students were accepted. As an exclusion criterion, it was requested that students with any diagnosis or treatment of personality or neurological alterations not participate. All students participated voluntarily and were taking undergraduate courses.

### Instruments

#### Self-Regulation

The *Short Self-Regulation Questionnaire (SSRQ)* was used to measure this variable (Brown, 1998; Brown et al., 1999). Its Spanish adaptation had been previously validated in Spanish samples (Pichardo et al., 2014; Garzón Umerenkova et al., 2017). Four factors are measured using a total of 17 items. The confirmatory factor structure is consistent (Chi-square = 250.83, df = 112, CFI = 0.95, GFI = 0.94, AGFI = 0.96, RMSEA = 0.059). Validity and reliability values (Cronbach’s alpha) were acceptable [total (*a* = 0.86; Omega = 0.843); goal setting-planning (*a* = 0.79; Omega = 0.784), perseverance (*a* = 0.78; Omega = 0.779), decision making (*a* = 0.72; Omega = 0.718), and learning from mistakes (*a* = 0.72; Omega = 0.722)], comparable to the English version. The scale contains statements such as: “I usually keep track of my progress toward my goals,” “When it comes to deciding about a change, I feel overwhelmed by the choice,” and “I learn from my mistakes.”

#### Self- vs. External Regulation of Behavior in Health

This *SRH-ERH Questionnaire* (de la Fuente, 2022) contains six subscales with six items each. Health-regulating aspects pertaining to the individual and to their context are assessed. Each item assesses either personal (internal) or contextual (external) aspects, whether regulatory, non-regulatory or dysregulatory. Some examples of each: (1) internal regulatory: I think consciously about my health needs, (2) external regulatory: the social context that I live in (family, environment, and friends) helps me plan my health-related behavior by setting goals and objectives; (3) internal non-regulatory (it is not necessary to make decisions in order to achieve changes in my health-related behaviors); (4) external non-regulatory: the social context that I live in (family, environment, and friends) gives me the idea that you do not need to make specific decisions to make changes in your health-related behaviors; (5) internal dysregulatory (it does not make sense to change your health-related behavior, if that takes away from your enjoyment and satisfaction); (5) external dysregulatory: the social context that I live in (family, environment, and friends) helps me enjoy myself to the fullest, it does not press me to change my health-related behavior, but rather to do what I feel like, if that makes me happy and live fully. The subscales in this instrument (de la Fuente, 2022) are: SRH (Self-Regulation health behavior), NRH (Non-Regulation or de-regulation health behavior), DRH (Dys-Regulation health behavior), ERH (External-Regulation Health behavior), ENRH (External Non-Regulation or De-regulation behavior behavior), EDRH (External Dys-Regulation Health behavior). Factor structure, as analyzed in this sample, is consistent [Chi-square = 1,348.005, df = 583, *p* < 0.001; Ch/df = 2.379; RMSR = 0.035; NFI = 0.967; RFI = 0.954; incremental fit index (IFI) = 0.902; TLI = 0.967; CFI = 0.978; RMSEA = 0.70]. Total reliability values were also acceptable (alpha total = 0.776). Subscale consistency was also acceptable: SRH = 0.847; NRH = 0.779; DRH = 0.769; ERH = 0.900; ENH = 0.761; EDH = 0.828.

#### Executive Function Difficulties

The Behavior Rating Inventory of Executive Function (BRIEF-A, Roth et al., 2005, 2014), adapted for university populations (de la Fuente, 2021), was used to assess EF difficulties (executive dysfunction). This questionnaire is a list of behaviors associated with EF impairment, self-reported by university students. The original version was published in order to study executive functioning in general populations, especially in pathologies such as attention deficit disorder with or without hyperactivity, learning disorders, pervasive developmental disorders, and disorders of neurological origin, such as traumatic brain injury, epilepsies (especially epilepsies with an epileptogenic focus in the temporal lobe), frontal tumors, cerebrovascular accidents, genetic syndromes, or cognitive impairment due to toxic exposure. This version contains 75 items grouped into 8 scales that measure different aspects of executive functioning difficulties: Inhibit, Shift, Emotional Control, Initiate, Working Memory, Plan/Organize, Organization of Materials, and Monitor. These scales are grouped into two general indices, Behavioral Regulation and Metacognition, and an overall score, the Global Executive Composite.

International guidelines for adaptation of psychological tests were followed for the adapting the BRIEF questionnaire to the Spanish university population (Muñiz et al., 2013). The values found for this sample were acceptable, both in construct validity (Chi-square = 81.550, df = 19, *p* < 0.001; Ch/df = 4.292; RMSR = 0.035; NFI = 0.944; RFI = 0.948; IFI = 0.957; TLI = 0.917; CFI = 0.956; RMSEA = 0.80), as well as in reliability (Cronbach’s alpha = 0.956; part 1 = 0.908, part 2 = 0.930).

#### Emotion Regulation Difficulties

These were assessed using the *Brief Difficulties in Emotion Regulation Scale*, DERS-16 (Bjureberg et al., 2016). The original DERS-36 self-report scale (Gratz and Roemer, 2004, 2008) contains 36 items that assess the individual’s typical levels of emotion dysregulation in six domains: non-acceptance of negative emotions, inability to engage in goal-directed behaviors when distressed, difficulties controlling impulsive behaviors when distressed, limited access to emotion regulation strategies perceived as effective, lack of emotional awareness, and lack of emotional clarity. The abbreviated version, DERS-16, contains 16 items that assess the following dimensions: non-acceptance of negative emotions (3 items), inability to engage in goal-directed behaviors when distressed (3 items), difficulties controlling impulsive behaviors when distressed (3 items), limited access to emotion regulation strategies perceived as effective (5 items), and lack of emotional clarity (2 items). In both versions, a Likert-type response is required, rating the degree to which each item is applicable, from 1 (almost never) to 5 (almost always). Total DERS-16 scores range from 16 to 80, where higher scores reflect greater levels of emotion dysregulation. The revalidation analyses in this sample showed adequate construct validity values (Chi-square = 26.054, df = 5, *p* < 0.001; Ch/df = 5.211; RMSR = 0.054; NFI = 0.954; RFI = 0.916; IFI = 0.962; TLI = 0.918; CFI = 0.962; RMSEA = 0.82), and reliability (Cronbach’s alpha = 0.888; part 1 = 0.803, part 2 = 0.831).

### Procedure

Student participation was on a voluntary basis, beginning with their agreement and signing of the informed consent statement, followed by anonymous completion of the scales on an online platform. The R&D Project was approved by the *Research Ethics Committee* of the University of Navarra (ref. 2018.170), and compliance with the deontological norms of psychology was assured. All databases are anonymized and protected by the Data Protection Law. The data collection server is located at (NETERRA DATACENTERS EUROPE^1^); where Mapache Software Europe fulfills the required handling and all assurances pertaining thereto. The Project IP^2^ is responsible for data protection and treatment.

### Data Analysis

Three types of analyses were conducted, using an *ex post* facto, transversal design (Ato et al., 2013). First, the quality of the data was explored by testing for outliers and missing cases. We tested for univariate outliers by calculating the typical scores of each variable, considering cases with *Z* scores outside the ±3 range to be potentially atypical (Tabachnick and Fidell, 2001). Atypical combinations of variables (atypical multivariate cases) were detected using the Mahalanobis distance (D2), a statistical measure of an individual’s multidimensional distance from the centroid or mean of the observations given (Lohr, 1999). In this way we detected instances with significant distance from the typical combinations of the set of variables. The literature recommends removing univariate and multivariate outliers, or reassigning them the nearest extreme score (Weston and Gore, 2006). The procedure was carried out using SPSS (v.26, IBM, Armonk, NY, United States), which provides a specific routine for missing values analysis that determines the magnitude of missing values and whether they occur in a systematic or random manner.

Assumptions related to sample size, independence of errors, univariate and multivariate normality, linearity, multicollinearity, recursion, and interval measurement level were also evaluated, and showed acceptable reliability levels. Regarding sample size, recommendations indicate including 10–20 cases per parameter, and at least 200 observations (Kline, 2005). Independence of errors means that the error term of each endogenous variable must not correlate with other variables. In order to test for univariate normality, we examined the distribution of each observed variable, and its asymmetry and kurtosis indices. Data transformation is recommended when asymmetry values are greater than 3 and kurtosis is greater than 10 (Kline, 2005). On the other hand, Mardia multivariate index values less than 70 indicate that distance from the multivariate normal is not a critical deterrent to this analysis (Rodríguez, 2011). Although level of interval measurement is one of the assumptions, variables measured at a nominal or ordinal level were sometimes used, as long as the score distribution, particularly of the dependent variables, was not markedly asymmetric (Weston and Gore, 2006).

The multicollinearity assumptions were tested through bivariate correlations; a correlation of 0.85 or higher would indicate non-fulfillment of this assumption. The model should be recursive: causal influences must be one-directional and not have retroactive effects. Finally, it is recommended that the instruments of measure show at least moderate reliability. This aspect was also fulfilled (see section “Instruments”). A power value of 0.80 was established as acceptable. The power of a statistical test relates to: (1) sample size *n*; (2) level of alpha significance: 5% was assumed, that is, a 95% confidence level (1-alpha); (3) effect size *d* or *r*: these measures indicate the relationship between variables (correlation coefficient). Low power may indicate a small sample size, a smaller alpha, or a small effect size, while the opposites may be indicated by high power.

Normal sample distribution was checked using the Kolmogorov–Smirnoff test for dependent variables, as a preliminary analysis. We also used the Hoelter Index to test for adequate sample size (Tabachnick and Fidell, 2001). In addition, we performed analyses of linearity and atypical values, missing and influential cases, as well as critical values of multivariate normality. Recommended values for the multivariate index of kurtosis, or Mardia coefficient, are less than 0.70 (Mardia, 1970).

For Hypothesis 1, Pearson bivariate correlations were carried out. For Hypothesis 2, we used multiple regression analysis. For Hypothesis 3, we used predictive analyses of structural equations, or SEM models. We followed Hu and Bentler’s (1999) recommendations, where a model shows adequate fit to the observed data if the ratio of the Chi-square to its degrees of freedom is less than five, RMSEA and SRMR values are <0.08, and NNFI (non-normed fit index), IFI and CFI are >0.95. For samples equal to or less than 250 participants, Hu and Bentler (1999) recommend using only the CFI and SRMR fit indices (not applicable in this case). The robust maximum likelihood method was used as an estimation method. This method allows the use of polychoric correlations, which are more suitable in variables with high normality indices and multivariate kurtosis, and a clearly ordinal nature [73]. Cronbach’s alpha was calculated in order to test the model’s total reliability, and the reliability of each of the proposed factor structures. For these analyses, we used SPSS 26 (IBM SPSS, 2019) for reliability, and AMOS v. 23 (Arbuckle, 2014) for the confirmatory factor analyses and the SEM model.

For the inferential hypothesis, Hypothesis 4, we initially calculated self-regulation and external regulation scores. In the first case, to calculate total personal regulation, we applied the summational formula of the values of self-regulation (+), non-regulation (−), and dysregulation (−), divided by three: (SR-ER-DR)/3, obtaining a weighted total score for each participant, ranging from 1 to −2.28. In the second case, to calculate external or contextual regulation, we applied the summational formula of the values of external regulation (+), external non-regulation (−), and external dysregulation (−), divided by three: (ER-ENR-EDR)/3, obtaining a continuous total score with a range between 1 and −2.17, for each participant. Subsequently, cluster analyses were performed to determine the central points and thus convert scores into low-medium-high groups for each type of regulation. The central points of the respective clusters were:

**Table:** 

	3. HIGH	2. MEDIUM	1. LOW
SR	−0.14	−0.72	−1.33
ER	0.32	−0.44	−1.13
			

Based on these central points, we calculated the distance between points and divided by two in order to establish cutoff points between the intervals:

**Table:** 

	3.0 HIGH	2.0 MEDIUM	1.0 LOW
SR	1 to −0.43	−0.044 to −1.02	−1.03 to −2.28
ER	1 to −0.06	−0.07 to −0.78	−0.079 to −2.13
			

With the scores now ordered on a range of 1 to 3, we calculated the average of the individual’s score and the regulatory score of their context, in each case. In this way we obtained a graded progression of five levels of combined personal and contextual regulation: 1.00 =

**Table:** 

Scores	Range						
SR	1.0	2.0	1.0	2.0	2.0	3.0	3.0
ER	1.0	1.0	2.0	2.0	3.0	2.0	3.0
AVERAGE	1.0	1.5	1.5	2.0	2.5	2.5	3.0

Based on the foregoing, this mean was taken as an IV, or heuristic on five levels, where significant between-group differences were confirmed using an ANOVA. Subsequently, ANOVAs and MANOVAs were carried out, taking EF and emotion regulation difficulties as dependent variables.

## Results

### Preliminary Results: Descriptive Results

The preliminary descriptive results showed acceptable fit and normality parameters (see Table 1).

**TABLE 1:** Normalized descriptive values of the sample.

Variable	Min.	Max.	Mean	(SD)	Error	Asymmetry	Error	Kurtosis	Error	Kolmogorov–Smirnov	Sig.
SR	2.06	4.47	3.4070	(0.02649)	0.02649	−0.089	0.142	−0.152	0.283	0.202	0.200
SRH	1.33	5.00	3.4840	(0.04142)	0.04142	−0.200	0.143	−0.213	0.284	0.169	0.200
NRH	1.00	4.67	2.3925	0.04421	0.04421	0.174	0.142	−0.592	0.284	0.213	0.200
DRH	1.00	4.50	2.4218	0.03983	0.03983	0.181	0.143	−0.184	0.285	0.248	0.177
ERH	1.00	5.00	3.4892	0.05085	0.05085	−0.230	0.142	−0.320	0.283	0.183	0.158
ENRH	1.00	4.50	2.3709	0.04572	0.04572	0.351	0.142	−0.391	0.284	0.242	0.200
EDRH	1.00	4.67	2.2144	0.04587	0.04587	0.291	0.142	−0.514	0.284	0.147	0.200
EF	1.07	3.81	2.2045	0.03317	0.03440	0.257	0.144	−0.555	0.287	0.115	0.171
ERD	1.35	4.28	2.6490	0.03440	0.03317	0.259	0.142	−0.297	0.283	0.169	0.200

*SR, Self-regulation; SRH, Self-regulation in Health; NRH, Non-regulation in Health; DRH, Dys-Regulation in Health; ERH, External-regulation in Health; ENRH, External Non-regulation in Health; EDRH, External Dys-Regulation in Health; EF, Executive Functions; ERD, Emotion regulation difficulties.*

### Linear Results: Association and Prediction

#### Self-Regulation, Executive Functions, and Emotion Regulation Difficulties

A significant negative association was found between total self-regulation and all the components of EF difficulties. The same was true for the components of both psychological constructs (see Tables 2, 3).

**TABLE 2:** Bivariate correlations between self-regulation (SR) and executive functions (EFs).

Variables	GOALS	PERSEVERANCE	DECISIONS	ERROR	SELF-REGULATION TOTAL
F1. INHIBITION	−0.259***	−0.222***	−0.153**	−0.285***	−0.339***
F2. FLEXIBILITY	−0.064	0.017	−0.332***	−0.182**	−0.187**
F3. CONTROL	−0.172**	−0.117*	−0.235**	−0.291**	−0.291**
F4. INITIATIVE	−0.388***	−0.268**	−0.263**	−0.450**	−0.450***
F5. MEMORY	−0.300***	−0.198**	−0.262**	−0.380**	−0.380**
F6. PLANNING	−0.381***	−0.274**	−0.278**	−0.442**	−0.442***
F7. ORGANIZATION	−0.111*	−0.160**	−0.169**	−0.246**	−0.246**
F8. MONITORING	−0.337***	−0.322***	−0.234**	−0.461**	−0.461***
D1. EMOTION	−0.343***	−0.136*	−0.282**	−0.452**	−0.452***
D2. COGNITIVE	−0.200**	−0.276**	−0.290***	−0.334**	−0.334***
EXECUTIVE DYSFUNCTION	−0.288**	−0.219**	−0.307***	−0.416**	−0.416***

*p < 0.05, **p < 0.01, ***p < 0.001.

**TABLE 3:** Bivariate correlations between self-regulation (SR) and emotion regulation difficulties (ERD).

Variables	GOALS	PERSEVERANCE	DECISIONS	ERROR	SELF-REGULATION TOTAL
F1. CLARITY	−0.164**	−0.063	−0.259***	−0.184	−0.232***
F2. STRATEGY	−0.149**	0.031	−0.277***	−0.218***	−0.209***
F3. ACCEPTANCE	−0.123*	0.022	−0.256***	−0.178**	−0.181*
F4. IMPULSIVITY	−0.235***	−0.118*	−0.272***	−0.238**	−0.305***
F5. GOALS	−0.075	0.054	−0.276***	−0.123*	−0.135*
EMOTION REGULATION DIFFICULTIES	−0.197**	−0.021	−0.354***	−0.247***	−0.280***

*p < 0.05, **p < 0.01, ***p < 0.001.

Regarding bivariate association relationships between SR and ERD, significant, inverse (negative) associations were found, both at a general level and with components of emotion regulation difficulty. Note that the greatest significant negative correlation was found between total SR and the component of Difficulty with Impulse Control, one of the emotion regulation difficulties (*r* = −0.305, *p* < 0.001). As for SR components, the clearest negative relationship was seen between decision making and ERD (*r* = −0.354, *p* < 0.001).

#### Self- vs. External-Regulation, Executive Dysfunction, and Emotion Regulation Difficulties

The association relationships between the components of SR-ER were differentially related to EFs. While SRH (self-regulated health behavior) and ERH (externally regulated health behavior) showed a significant, positive relationship, non-regulated behavior, and context (NRH and ENRH) were shown to have a significant, moderate relationship (*r* = 0.210, *p* < 0.001; *r* = 0.352, *p* < 0.001). A positive direction was also observed in the significant positive association with dysregulatory health behavior (DRH; *r* = 0.292, *p* < 0.001) and dysregulatory health context (EDRH; *r* = 0.342, *p* < 0.001). The most consistent association observed was between the cognitive dimension and its factors, where higher association values went to subjects’ lack of initiative (*r* = 0.436, *p* < 0.001) and lack of monitoring (*r* = 0.436, *p* < 0.001), respectively; and in a non-regulatory context, lack of monitoring and organization (*r* = 0.388, *p* < 0.001) and lack of inhibition (*r* = 0.372, *p* < 0.001). Also important, from the dysregulatory context, was the positive association with lack of monitoring (*r* = 0.359; *p* < 0.001) and lack of planning (*r* = 0.329; *p* < 0.001). In complementary fashion, in all NRH and DRH behaviors, the strength of association was greatest with the cognitive dimension of EF (see Tables 4, 5).

**TABLE 4:** Bivariate correlations between self vs. external regulation (SR-ER) and difficulties inherent to executive functions (EFs).

Variables	SRH	NRH	DRH	ERH	ENRH	EDRH
F1. INHIBITION	−0.206***	0.428***	0.278**	−0.081	0.372***	0.322***
F2. FLEXIBILITY	−0.108*	0.192**	0.158*	−0.054	0.206**	0.169*
F3. CONTROL	−0.161**	0.283***	0.223**	−0.048	0.192*	0.240**
F4. INITIATIVE	−0.249***	0.436***	0.252***	−0.232**	0.341***	0.311**
F5. MEMORY	−0.244***	0.397***	0.195*	−0.156*	0.327***	0.275**
F6. PLANNING	−0.247***	0.395***	0.247***	−0.223**	0.280**	0.329**
F7. ORGANIZATION	−0.238***	0.393***	0.243***	−0.107*	0.287**	0.275**
F8. MONITORING	−0.299**	0.398***	0.325***	−0.257**	0.388***	0.359***
D1. EMOTION	−0.309***	0.477***	0.291***	−0.230**	0.371***	0.354***
D2. COGNITIVE	−0.192**	0.366***	0.268**	−0.071	0.311**	0.298**
EXECUTIVE DYSFUNCTION	−0.268***	0.447***	0.292***	−0.162**	0.352***	0.342***

*SR, Self-regulation; SRH, Self-Regulation in Health; NRH, Non-Regulation in Health; DRH, Dys-Regulation in Health; ERH, External-Regulation in Health; NRH, External Non-Regulation in Health; EDRH, External Dys-Regulation in Health.*

*p < 0.05, **p < 0.01, ***p < 0.001.

**TABLE 5:** Bivariate correlations between self vs. external regulation (SR-ER) and difficulties inherent to executive functions (EFs).

Variables	SRH	NRH	DRH	ERH	ENRH	EDRH
F1. CLARITY	−0.180**	0.210**	0.174*	0.044	0.132*	0.151*
F2. STRATEGY	−0.114*	0.224**	0.190*	−0.087	0.259**	0.252**
F3. ACCEPTANCE	−0.112*	0.171**	0.109*	−0.081	0.189*	0.199*
F4. IMPULSIVITY	−0.153**	0.249***	0.275**	−0.092	0.221**	0.243**
F5. GOALS	−0.045	0.062	0.051	−0.025	0.077	0.148*
EMOTION REGULATION DIFFICULTY	−0.161**	0.241***	0.210**	−0.060	0.228**	0.259**

*SR, Self-regulation; SRH, Self-Regulation in Health; NRH, Non-Regulation in Health; DRH, Dys-Regulation in Health; ERH, External-Regulation in Health; NRH, External Non-Regulation in Health; EDRH, External Dys-Regulation in Health.*

*p < 0.05, **p < 0.01, ***p < 0.001.

The association trend was similar with Emotion regulation difficulties (ERD) and its components. Also, with less associative strength, personal, and contextual behavioral characteristics (NRH, DRH, ENRH, and EDRH) were positively associated with ERD.

### Linear Prediction Results

#### Preliminary Analysis: Prediction of Self-Regulation From Self-Regulation–External Regulation Components

Preliminary prediction analysis showed a significant linear model [*F*(6,280) = 13.144, *p* < 0.001; adjusted *R*^2^ = 0.203] where the factors “self-regulation” (*B* = 0.339, *p* < 0.001), “non-regulation” (*B* = −0.0.99, *p* < 0.155) and “dys-regulation” (*B* = −0.126, *p* < 0.037) were shown to be differential predictors of general SR. The factors ER (*B* = 0.0.29, *p* < 0.649), ENR (*B* = −0.0.66, *p* < 0.353) and EDR (*B* = 0.006, *p* < 0.932) did not present significant predictions.

#### Prediction of Executive Dysfunctions and Emotion Regulation Difficulties From Self-Regulation Components

The first prediction analysis showed a significant linear model [*F*(4,282) = 17.976, *p* < 0.001; adjusted *R*^2^ = 0.192] where the factors of “goals” (*B* = −0.115, *p* < 0.07), “decision making” (*B* = −0.230, *p* < 0.001) and “learning from mistakes” (*B* = −0.271, *p* < 0.001) appeared as significant negative predictors of EFs. Note that the percentage of explained variance is less than in the following case.

The second prediction analysis showed a significant linear model [*F*(4,293) = 16.595, *p* < 0.001; adjusted *R*^2^ = 0.176 (17% of the explained variance)] where the factors of “decision making” (*B* = −0.316, *p* < 0.001) and “learning from mistakes” (*B* = −0.247, *p* < 0.001) appeared as significant negative predictors, while “perseverance” was a significant positive predictor (*B* = 0.163, *p* < 0.01) of emotion regulation difficulties.

#### Prediction of Executive Dysfunctions and Emotion Regulation Difficulties From Self-Regulation–External Regulation Components

The first prediction analysis showed a significant linear model [*F*(6,274) = 17.273, *p* < 0.001; adjusted *R*^2^ = 0.259 (25.9% of the explained variance)] where “self-regulation, SR” (*B* = −0.114, *p* < 0.08) was a marginally significant negative predictor, while “non-regulation, NR” (*B* = 0.270, *p* < 0.001), “external non-regulation, ENR” (*B* = 0.136, *p* < 0.05) and “external dysregulation, EDR” (*B* = 0.167, *p* < 0.01) were significant positive predictors of EFs.

The second prediction analysis showed a significant linear model [*F*(6,280) = 6.122, *p* < 0.001; adjusted *R*^2^ = 0.097 (9.7% of the explained variance)] where “SR” (*B* = −0.118, *p* < 0.05) was shown to be a significant negative predictor, while “dysregulation, DR” (*B* = 0.162, *p* < 0.05) was a significant positive predictor of emotion regulation difficulties.

#### Predicting Emotion Regulation Difficulties From Components of Executive Dysfunctions

The prediction analysis showed a significant linear model [*F*(2,284) = 59,275, *p* < 0.001; *R*^2^ = 0.290 (29% of the explained variance)] where the Emotional dimension of EFs (D1) was a significant positive predictor of Emotion regulation difficulties (*B* = 0.544, *p* < 0.001), while the Cognitive factor of EFs did not show predictive ability.

#### Structural Prediction

Of the models tested, the second fulfills the statistical parameters required for empirical fit (see Table 6).

**TABLE 6:** Statistical parameters of structural models.

Models	Type factors	Chi-square	Degrees of freedom	*p*<	CMIN/DF	TLI	RFI	IFI	TLI	CFI	RMSEA	HO 0.05	HO 0.01
Model 1	4 F	826,600	(299–75): 224	0.001	3,690	0.756	0.699	0.809	0.761	0.806	0.095	93	99
Model 2*	4 F	827,467	(299–73): 226	0.001	3,361	0.914	0.901	0.909	0.914	0.906	0.083	94	100

*L, learning process; T, teaching process.*

*Selected models.

**FIGURE 1:**
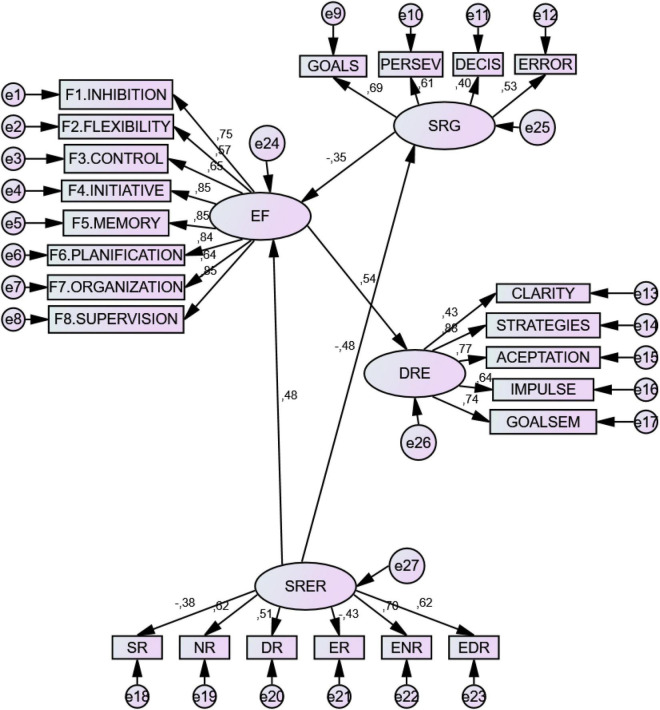
Structural predictive model of relationships. EF, Executive Dysfunction; SRER, Self-regulation vs. External regulation; ERD, Emotion Regulation Difficulties; SR, Self-regulation; SRH, Self-Regulation in Health; NRH, Non-Regulation in Health; DRH, Dys-Regulation in Health; ERH, External-Regulation in Health; NRH, External Non-Regulation in Health; EDRH, External Dys-Regulation in Health.

Model 3 reflected how SR-ER factors were negative predictors of *Self-regulation* (SR), and positive predictors of Executive Function (EF) and Emotion Regulation Difficulties (ERD). Complementarily, self-regulation (SR) negatively predicted Emotion Regulation Difficulties (ERD) and Executive Function (EF). Finally, *Executive Dysfunction* (EF) difficulties were positively predictive of Emotion Regulation Difficulties (ERD) (see Table 7 and Figure 1).

**TABLE 7:** Total, indirect, and direct effects of the variables in this study, and 95% bootstrap confidence intervals (CI).

Predictive variable	Criterion variable	Total effect	CI (95%)	Direct effect	CI (95%)	Indirect effect	CI (95%)	Results, effects	CI (95%)
SRER→	SR	−0.476	[−0.27, −52]	−0.476	[−0.27, −52]	0.00	[−0.03, 0.02]	Direct only	[−0.27, −52]
SRER→	EF	0.649	[0.45, 0.76]	0.482	[0.56, 38]	0.166	[0.22, 0.12]	Partial mediation	[0.22, 0.12]
SRER→	ERD	0.351	[0.43, 0.27]	0.00	[−0.15, 0.18]	0.351	[0.43, 0.27]	Full mediation	[0.43, 0.27]
SR	EF	−0.350	[−31, −0.37]	−0.350	[−31, −0.37]	0.00	[−0.03, 0.04]	Direct only	[−31, −0.37]
SR	ERD	−0.189	[−0.20, −0.28]	0.00	[−0.03, 0.04]	−0.189	[−0.20, −0.28	Full mediation	[−0.20, −0.28]
EF→	ERD	0.541	[0.48, 62]	0.541	[0.48, 62]	0.00	[−0.03, 0.02]	Direct only	[−0.03, 0.02]

*CI, confidence interval. Bootstrapping sample size = 298.*

Figure 1 shows predictive relationships of the model. The latent variable SR-ER positively predicts EF (*B* = 0.48). The factors of non-regulation (NR), dysregulation (DR), external non-regulation (ENR), and external dys-regulation (EDR) have positive predictive weight in the configuration of the model, while self-regulation (SR) and external regulation (ER) have negative weight. The latent variable SR-ER also negatively predicts SR (*B* = −0.48), and SR negatively predicts EF (*B* = −0.35). Finally, the latent variable EF positively predicts ERD (*B* = 0.54) (see Figure 1).

### Inferential Results

#### Effect of the Level of Executive Dysfunctions on Self-Regulation, and on Self-Regulation vs. External Regulation

##### Effect on Self-Regulation

There was a significant statistical main effect of the level of EFs on the variable self-regulation (SR) [*F*(2,284) = 24.065, *p* < 0.001; eta^2^ = 0.145; 3 > 2 > 1, *p* < 0.001]. Levene’s test of equality of error variances, based on the mean, showed no significant between-group differences [*Levene* (2,284) = 0.351, *p* < 0.704] (see Table 8).

**TABLE 8:** Effect of low–medium–high levels of the independent variable executive functions (EF) on SR.

Level of the independent variable EF	Mean of the dependent variable SR	(SD)	*Post hoc* (Scheffé)
1 (*n* = 97) low	3.6193	(0.44024)	1 > 2, 3***
2 (*n* = 121) medium	3.3859	(0.39829)	3 > 2 > 1***
3 (*n* = 69) high	3.1630	(0.43186)	3 < 2, 1***
Total	3.4112	(0.45363)	

*EF, Executive Function; SR, Self-Regulation.*

***p < 0.001.

##### Effect on Self-Regulation vs. External-Regulation

Box’s *M*, a preliminary test for matrix equality, showed no significant between-group differences [*F*(42,159347) = 1.526, *p* < 0.716]. There was a significant statistical main effect of the level of EFs on the variable self- vs. external regulation (SR-ER) [*F*(2,284) = 7.124, *p* < 0.001; eta^2^ = 0.145; 3 > 2 > 1, *p* < 0.001]. Note the greater discriminant strength in the factors NR, ENR, DR, and EDR (see Table 9).

**TABLE 9:** Effect of low–medium–high levels of the independent variable executive functions (EF) on SR-ER.

Dependent variables	1. Low EF (*n* = 94)	(SD)	2. Medium EF (*n* = 119)	(SD)	3. High EF (*n* = 68)	(SD)	Mean EF (*n* = 281)	(SD)	*F*(2,278)	*Post hoc*
SR	3.702	(0.645)	3.420	(0.717)	3.269	(0.700)	3.478	(0.708)	8.481***	1 > 2, 3***
NR	2.039	(0.667)	2.392	(0.693)	2.860	(0.713)	2.387	(0.753)	27.953***	3 > 2 > 1***
DR	2.164	(0.612)	2.451	(0.644)	2.698	(0.679)	2.415	(0.679)	13.591***	3, 2 > 1***
ER	3.586	(0.834)	3.484	(0.923)	3.279	(0.763)	3.469	(0.862)	2.569*	1, 2 > 3***
ENR	2.037	(0.692)	2.375	(0.742)	2.777	(0.730)	2.359	(0.772)	20.692***	3 > 2 > 1***
EDR	1.9663	(0.784)	2.145	(0.709)	2.639	(0.728)	2.205	(0.781)	17.008***	3 > 2, 1***

*SR, Self-regulation; NR, Non-Regulation; DR, Dys-regulation; ER, External-Regulation; ENR, External Non-Regulation; EDR, External Dys-Regulation.*

*p < 0.05, ***p < 0.001.

##### Effect on Emotion Regulation Difficulties

Levene’s test of equality of error variances, based on the mean, showed no significant between-group differences [*L*(2,284) = 1.216, *p* < *0.298*]. There was a significant statistical main effect of the level of EFs on the variable Emotion regulation difficulties (ERD) [*F*(2,278) = 35.202, *p* < 0.001; *R*^2^ = 0.199; 3 > 2 > 1, *p* < 0.001]. This main effect was consistent both for the total score and for the factors. In this case, the effect on factor 2 (lack of emotion regulation strategies) and factor 4 (lack of impulse control) stand out as having the greatest main effect (see Table 10).

**TABLE 10:** Effect of low–medium–high levels of the independent variable executive dysfunctions (EF) on emotion regulation difficulties (ERD).

Dependent variables	1. Low EF (*n* = 94)	(SD)	2. Medium EF (*n* = 119)	(SD)	3. High EF (*n* = 68)	(SD)	Mean EF (*n* = 281)	(SD)	*F*(2,278)	*R* ^2^	*Post hoc*
ERD total	2.304	(0.464)	2.756	(0.533)	2.930	(0.533)	2.645	(0.568)	35.202***	0.199	1 < 2 < 3***
F1. Clarity	2.030	(0.803)	2.324	(1.03)	2.608	(1.12)	2.419	(1.02)	11.409***	0.074	1 < 2 < 3***
F2. Strategies	1.9567	(0.629)	2.6066	(0.778)	2.994	(0.746)	2.480	(0.826)	44.631***	0.239	1 < 2 < 3***
F3. Acceptance	2.010	(0.849)	2.650	(0.954)	2.903	(0.918)	2.494	(0.977)	22.400***	0.136	1 < 2 < 3***
F4. Impulse	1.793	(0.706)	2.294	(0.872)	2.821	(0.860)	2.252	(0.901)	32.163***	0.185	1 < 2 < 3***
F5. Goals	2.611	(0.903)	3.159	(0.932)	3.125	(0.864)	2.966	(0.937)	11.258***	0.073	1 < 2 < 3***

*ERD, Emotion Regulation Difficulties; F1, lack of emotional clarity; F2, emotion management strategies; F3, lack of acceptance; F4, lack of impulse control; F5, Difficulty in emotional goals.*

***p < 0.001.

#### Effects of the Combined Self-Regulation–External Regulation Level on Executive Functions and on ERD

##### Preliminary Checks for Group Adequacy

The MANOVA used to test the adequacy of the groups showed a significant main effect of the SR-ER combination on the dependent variables analyzed [*F*(8,566) = 49.846, *p* < 0.001, *R*^2^ = 0.413; power = 1.0], with a greater significant effect on the variable of context regulation (ERcurve). Subsequent analyses revealed the expected significant differences between groups (see *post hoc* in the table). Box’s *M* test for equality of covariance matrices also showed lack of equality between the group variances [*M* = 68.234; *F*(12,6272.902) = 7.44, *p* < 0.10] (see Table 11).

**TABLE 11:** Effect of the combined SR-ER levels on the continuous dependent variables SR and ER.

	Level 1.0. (*n* = 19)	Level 1.5. (*n* = 70)	Level 2.0. (*n* = 107)	Level 2.5. (*n* = 80)	Level 3.0. (*n* = 9)	TOTAL (*n* = 285)	*F*(4,483)	*Post hoc*
SRCURVE	−1.277 (0.178)	−1.001 (0.353)	−0.762 (0.493)	−0.597 (0.268)	−0.222 (0.209)	−0.798 (0.438)	23.456	3.00 < 2.50 < 2.00 < 1.50 < 1.00
ERCURVE	−1.129 (0.222)	−0.839 (0.455)	−0.344 (0.537)	0.1382 (0.447)	0.222 (0.220)	−0.373 (0.630)	57.749	3.00 < 2.50 < 2.00 < 1.50 < 1.00

##### Effects on Executive Dysfunctions

The first ANOVA, referring to the effect of SR-ER combinations on total EF score, showed a significant main effect [*F*(4,280) = 25.006, *R*^2^ = 0.365, power = 1.0]; Levene’s test of equality of error variance, based on means, also showed an absence of significant between-group differences [*L*(4,280) = 1.287, *p* < 0.275]. The second MANOVA, referring to the EF dimensions, showed another significant main effect [*F*(8,560) = 13.237, *R*^2^ = 0.159, power = 1.0]. The MANOVA performed with respect to the EF factors also showed a significant main effect [*F*(32,1104) = 3.765; *p* < 0.001, *R*^2^ = 0.098; power = 1.0]. Box’s Test of equality of covariance matrices showed a similarity of covariance matrices [Box’s *M* = 325.351; *F* = 1.847; df1 = 144; df2 = 4,346.966; *p* < 0.10]. Levene’s test of equality of error variances, based on means, for each factor, also showed an absence of significant between-group differences [*L*(4,280) = between 0.280 and 1.287, *p*<, between 0.230 and 0.841]. Note that the greatest main effect was on *cognitive* EFs, that is, lack of initiative, planning and organization. The greatest effect in *emotional* EFs refers to inhibition difficulty (see Table 12).

**TABLE 12:** Effect of the SR-ER combination levels on the dependent EF (Executive Dysfunction) variables.

	Level 1.0. (*n* = 19)	Level 1.5. (*n* = 70)	Level 2.0. (*n* = 107)	Level 2.5. (*n* = 80)	Level 3.0. (*n* = 9)	TOTAL (*n* = 285)	*F*(4,280)	*R* ^2^	*Post hoc* (Scheffé)
EF total	2.716 (0.492)	2.548 (0.591)	2.201 (0.499)	1.836 (0.428)	1.609 (0.504)	2.206 (0.584)	25.006***	0.365	1.0 > 1.5 > 2.0 > 2.5 > 3.0***
D1. COGNITIVE	2.751 (0.275)	2.577 (0.604)	2.240 (0.593)	1.733 (0.248)	1.698 (0.591)	2.197 (0.655)	28.842***	0.292	1.0 > 1.5 > 2.0 > 2.5 > 3.0***
D2. EMOTION	2.680 (0.474)	2.489 (0.624)	2.162 (0.580)	1.939 (0.515)	1.719 (0.545)	2.214 (0.594)	15.072***	0.177	1.0 > 1.5 > 2.0 > 2.5 > 3.0***
F1. INHIBITING	2.431 (0.684)	2.291 (0.791)	1.822 (0.592)	1.506 (0.493)	1.411 (0.430)	1.876 (0.702)	19.988***	0.222	1.0 > 1.5 > 2.0 > 2.5 > 3.0***
F2. FLEXIBILITY	2.940 (0.574)	2.655 (0.792)	2.475 (0.631)	2.279 (0.660)	2.347 (0.777)	2.491 (0.703)	5.092***	0.068	1.0 > 1.5 > 2.0 > 2.5 > 3.0***
F3. MONITORING	2.669 (0.616)	2.511 (0.792)	2.188 (0.742)	2.031 (0.754)	2.000 (0.787)	2.274 (0.786)	7.546***	0.097	1.0, 1.5 > 2.0 > 2.5, 3.0***
F4. INITIATING	2.684 (0.720)	2.555 (0.702)	2.160 (0.681)	1.714 (0.500)	1.680 (0.603)	2.151 (0.725)	20.724***	0.228	1.0, 1.5 > 2.0 > 2.5, 3.0***
F5. MEMORY	2.689 (0.641)	2.545 (0.674)	2.257 (0.736)	1.771 (0.603)	1.533 (0.644)	2.203 (0.746)	16.309***	0.189	1.0, 1.5 > 2.0 > 2.5, 3.0***
F6. PLANNING	2.644 (0.591)	2.509 (0.742)	2.316 (0.745)	1.87 (0.686)	1.780 (0.538)	2.270 (0.766)	19.958***	0.222	1.0, 1.5 > 2.0 > 2.5, 3.0***
F7. ORGANIZATION	3.017 (0.881)	2.611 (0.873)	2.269 (0.935)	1.600 (0.793)	1.555 (0.803)	2.193 (0.899)	17.159***	0.197	1.0 > 1.5 > 2.0 > 2.5, 3.0***
F8. MONITORING	2.727 (0.608)	2.464 (0.627)	2.199 (0.584)	1.800 (0.511)	1.652 (0.681)	2.170 (0.649)	18.784	0.212	1.0 > 1.5 > 2.0 > 2.5 > 3.0***

***p < 0.001.

##### Effects on Emotion Regulation Difficulties

The ANOVA referring to the effect of SR-ER combination levels on total ERD score showed a significant main effect [*F*(4,286) = 8.719, *R*^2^ = 0.109, power = 0.99]; Levene’s test of equality of error variances, based on means, also showed an absence of significant between-group differences [*L*(4,286) = 1.085, *p* < 0.364].

The MANOVA referring to the effect of SR-ER combination levels on ERD dimensions, showed another significant main effect [*F*(20, 1140) = 3.227, *R*^2^ = 0.054, power = 1.0]. Box’s test of equality of covariance matrices showed similarity among them [Box’s *M* = 93.740; *F* = 1,398; df1 = 60; df2 = 4717,316; *p* < 0.10]. Levene’s test of equality of error variances, based on means, for each factor, also showed an absence of significant between-group differences [*L*(4,286) = between 0.058 and 0.090; *p* < between 0.121 and 0.927] (see Table 13).

**TABLE 13:** Effect of the SR-ER combination levels on the dependent ERD variables (Emotion Regulation Difficulties).

	Level 1.0. (*n* = 19)	Level 1.5. (*n* = 70)	Level 2.0. (*n* = 107)	Level 2.5. (*n* = 80)	Level 3.0. (*n* = 9)	TOTAL (*n* = 285)	*F*(4,280)	*R* ^2^	*Post hoc*
ERD	2.890 (0.561)	2.788 (0.732)	2.528 (0.680)	2.250 (0.660)	2.061 (0.870)	2.526 (0.770)	8.719***		1.0, 1.5 > 2.0 > 2.5, 3.0***
F1. CLARITY	2.809 (0.679)	2.760 (1.21)	2.394 (0.098)	2.123 (0.831)	1.833 (1.08)	2.421 (1.02)	5.505***	0.072	1.0, 1.5 > 2.0 > 2.5 > 3.0***
F2. STRATEGIES	2.961 (0.755)	2.816 (0.829)	2.418 (0.768)	2.187 (0.752)	2.177 (1.03)	2.483 (0.826)	8.512***	0.106	1.0, 1.5 > 2.0 > 2.5, 3.0***
F3. ACCEPTANCE	2.825 (0.711)	2.676 (0.944)	2.596 (1.02)	2.156 (0.899)	2.111 (1.05)	2.494 (0.976)	4.490***	0.059	1.0 > 1.5 > 2.0 > 2.5, 3.0***
F4. IMPULSE	2.841 (0.820)	2.591 (0.887)	2.204 (0.915)	1.971 (0.773)	1.703 (1.04)	2.264 (0.910)	8.812***	0.103	1.0 > 1.5 > 2.0 > 2.5, 3.0***
F5. GOALS	3.015 (0.702)	3.098 (0.929)	3.027 (0.925)	2.814 (0.961)	2.481 (1.01)	2.967 (0.969)	1.656	0.023	1.0, 1.5 > 2.0 > 2.5, 3.0***

#x002A;**p < 0.001.

## Discussion

This research aimed to establish the predictive relationships between a molecular construct (SR) and a molar construct (SR-ER) with respect to a microanalytical (EF) construct and a clinical correlate (ERD), in order to provide evidence of the predictive value of these variables. The results presented here uphold the proposed relationships overall.

Regarding the association Hypothesis (1), the proposed significant relationships were found: there is an inverse relationship between SR and EF difficulties, as has been previously and sufficiently documented (Baumeister and Heatherton, 1996). SR has been shown to be widely associated with personal well-being and healthy behaviors (Morosanovaa et al., 2021a), as well as with successful complex learning (Morosanovaa et al., 2021b).

One novel result pertains to the fact that the non-regulatory and dysregulatory contexts were positively associated with EF and ERD. This result is important because: (1) it lends support to and broadens the conceptualization of EF, as a construct associated with non-regulatory behavior (less studied) and dys-regulatory behavior (Beheshti et al., 2020); (2) of particular importance, this result documents the role of the non-regulatory and dys-regulatory context in association with the level of EFs, something that has not been addressed in classic conceptualizations (Diamond, 2013); (3) Finally, this result incorporates the specific role of a lack of regulation and of dysregulation into explanatory models of EF. Present within subjects and also in the context in which they develop, these aspects in combination help to explain the behavioral dysfunctions that are typical of executive dysfunction (Munakata and Michaelson, 2021). Correlational studies have documented reliable links between children’s environments and their outcomes in multiple domains. For example, inconsistent discipline from caregivers predicts higher negative affect and behavioral problems in children (Doan and Evans, 2020), and regular family routines (such as consistent meal- and bedtimes) are associated with positive developmental outcomes (Fiese et al., 2006). Better childhood EF has been related to more positive parenting (e.g., warmth and responsiveness), less negative parenting (e.g., control and intrusiveness), and parenting that is more cognitive (e.g., autonomy support and scaffolding) (Valcan et al., 2018). When children have more unstructured time in their daily life for using engaging EFs, better self-directed executive functioning is displayed on laboratory tasks (Barker et al., 2014). By contrast, when parents and other adults in children’s lives show unpredictable and unreliable behavior, this is associated with poorer executive functioning on tasks regarding delayed gratification and temporal discounting (Mauro and Harris, 2000). Household chaos is also associated with poorer executive functioning in children (Schmidt et al., 2015; Suor et al., 2017; Andrews et al., 2021). Cultures also vary in how they relate to EFs, the value they associate with them, and their tendency to engage them (Yanaoka and Saito, 2021). Finally, as expected, a positive association was found between EF and ERD, showing that executive dysfunction is associated with emotional regulatory dysfunction (Eisenberg et al., 2010).

The results above were qualified by the linear and structural prediction Hypotheses (2 and 3). Thus, the components of SR proved to be negative predictors of the total EF score (Hofmann et al., 2012). SR-ER factors were differentially predictive of EF factors; while SR and ER factors negatively predicted EF; NR-ENR and DR-EDR were positive predictors, as in other previous findings (Bernier et al., 2010; Diamond, 2016).

Also, the EF (executive dysfunction) components proved to be positive predictors of Emotion Regulation Difficulties (ERD), especially those corresponding to the behavior regulation dimension. Although this study uses a normalized sample and ADHD students did not participate, some of these results could help us understand other relationships found in previous research. A relationship has been observed between *Attention-Deficit/Hyperactivity Disorder (ADHD*), as a case of *executive dysfunction*, and difficulties with regulating emotions, with certain conclusive results. First, emotion dysregulation in ADHD persists throughout one’s lifespan and is a major factor contributing to impairment. Second, this dysregulation may be due to deficits in how one orients to, recognizes, and/or assigns attention to emotional stimuli; such deficits involve dysfunction within a striato-amygdalo-medial prefrontal cortical network. Third, while current treatments often improve emotion dysregulation, a focus on this combination of symptoms reframes clinical questions and could stimulate new therapeutic approaches. Emotion dysregulation and ADHD are correlated but are distinct dimensions. Emotion dysregulation is a core aspect of an ADHD diagnosis; the combination constitutes a nosological entity, distinct from both ADHD and emotional dysregulation alone (Shaw et al., 2014; Villemonteix et al., 2014).

Regarding the inferential Hypotheses (4), it was possible to show that the level of EFs determined the level of the remaining variables. Complementarily, the five combination levels of internal and external regulation (SR-ER) were significant negative determinants of EF and of the degree of emotion regulation difficulty (ERD), although differentially. The combination of lower SR-ER levels determined higher levels of EF and emotion regulation difficulty, and vice versa, along a gradient. These results resemble others obtained in our previous investigations (de la Fuente, 2017; de la Fuente et al., 2021a,b), but they must be revalidated by new research studies as well.

### Evidence

Based on the results given, it is possible to place the EF construct in direct relationship to the SR vs. ER theoretical model. At the subject level, the SR variable is the inverse of the construct; that is, a high score in executive dysfunction leads to a low score in SRH, while NRH and DRH are high; this allows us to establish a classification continuum of university students in their individual health behavior. At the context level, ER also appears as the inverted side of the construct; that is, a high score in executive dysfunction leads to a low score in ERH, and at the same time, a high score in ENRH and EDRH, allowing us to understand a classification continuum of university students’ context, to the extent that it promotes health behaviors. Finally, this research has made it possible to establish an averaged combination continuum of the above variables in a five-level combination heuristic, which accounts for the possible combinations between personal and contextual factors, and their effect on the level of emotion regulation difficulty (ERD).

### Limitations and Research Prospects

Limitations due to sample size and invitation to respond may have led to a selection bias. Specifically, there is a clear limitation regarding gender: the sample contains a much higher percentage of women (63.5%) than men (36.5%). In addition, the fact that these results come from a university sample does not allow extrapolation to other stages of education. At the same time, this may also be considered a goodness: this analysis addresses the question of EF at university level, where there has been little research on this construct. Future research should establish whether this theoretical model can explain and account for other difficulties inherent to students at this stage of education, given the importance of preventive and health promotion programs at this stage of human development.

One prospect of interest, for an adequate connection between the different levels of analysis of self-regulation behavior (microanalysis, molecular, and molar) is to complement the analysis of relationships focused exclusively on personal characteristics, by integrating the role of contextual variables. This is especially relevant when explaining delinquency or sanctionable behaviors, so as not to minimize contextual explanatory variables (Coenen et al., 2021).

### Implications for the Psychology Profession

There are several professional and practical implications of this research: (1) The concept of executive dysfunction should be categorized in the proposed SR-NR-DR continuum by the SR vs. ER Theory model (2017, 2021). (2) Assessment of this construct, using the new SR-ER scale, gives us access to information from the personal and contextual regulatory domains, helping us understand that there are personal and contextual factors in protection and risk of dysregulation. (3) Psychological intervention should focus not only on moving the individual from dysregulatory to self-regulatory behavior, but also on moving from a dysregulatory to an externally regulatory context. From the standpoint of educational psychology, interventions can help toward a more regulatory design of formal, non-formal and informal education or teaching-learning contexts. In clinical and health psychology, they can contribute to increasing external regulation through contextual signals that promote health behaviors and satisfaction in the health context, and minimize dysregulatory contexts. In social psychology, progress can be made in helping organizations to avoid dys-regulatory contexts, and to promote and aid self-regulation in the organization. Such intervention can be key in enabling people with problems in EF to work and perform better, as well as in facilitating more adaptive behavior in different behavioral contexts. In short, it is time to complement the microanalysis (neurological) and molecular (clinical) models of executive dysfunction with molar (contextualized) models that allow us to analyze the role of a dysregulatory context in this behavioral problem.

## Conclusion

The model proposed in the SRL vs. ERL Theory (2017), referring to the Self-Regulation-External Regulation construct, can be a good analysis heuristic for college students’ learning and health behaviors, especially if they have any specific EF or emotion regulation difficulties. We must define what level of analysis of learning processes we want to carry out, and, based on this decision, choose the appropriate model. If one’s intent is to understand the specific cognitive mechanisms involved in health behaviors, with a high degree of concreteness, it would be appropriate to work in the domain of micro-analysis: the analysis of EFs (Brown and Landgraf, 2010; Diamond and Lee, 2011; Barkley, 2012; Climent-Martínez et al., 2014). If one’s objective is to understand the strategies involved in an important learning task, from a clinical perspective, a molecular level heuristic model is a better choice, i.e., general self-regulation (SR) (Liew, 2011; Miller et al., 2011; Ahmed et al., 2019). If one desires to understand difficulty, including the role of context, it seems more useful to adopt a molar level of analysis (SR vs. ER) (Doebel, 2020; Tzuriel, 2021). For all the above reasons, it is essential that we assign models to their proper scope and their object of study, understanding their strengths and limitations. Otherwise, it will be difficult for us to integrate the different existing levels in a coherent analysis of the numerous contributions regarding EF, and to train educators (family members and teachers) in these aspects (Fitzpatrick et al., 2020).

## Data Availability Statement

The raw data supporting the conclusions of this article will be made available by the authors, without undue reservation.

## Ethics Statement

This study was reviewed and approved by Comité de Ética de la Universidad de Navarra, ref. 2018.170. The patients/participants provided their written informed consent to participate in this study.

## Author Contributions

JF and JM-V: rationale, design, data analysis, and writing. MP-B: review and initial data adjustment. FP-S, MV-M, and MA-R: data collection. All authors contributed to the article and approved the submitted version.

## Conflict of Interest

The authors declare that the research was conducted in the absence of any commercial or financial relationships that could be construed as a potential conflict of interest.

## Publisher’s Note

All claims expressed in this article are solely those of the authors and do not necessarily represent those of their affiliated organizations, or those of the publisher, the editors and the reviewers. Any product that may be evaluated in this article, or claim that may be made by its manufacturer, is not guaranteed or endorsed by the publisher.

## Footnotes

1
https://www.icdsoft.com/en/datacenters#europe

2
www.inetas.net
